# Novel Solvent–Latex Mixing: Thermal Insulation Performance of Silica Aerogel/Natural Rubber Composite

**DOI:** 10.3390/gels8010007

**Published:** 2021-12-22

**Authors:** Chayanan Boonrawd, Supan Yodyingyong, Lazhar Benyahia, Darapond Triampo

**Affiliations:** 1 Department of Chemistry and Center of Excellence for Innovation in Chemistry, Faculty of Science, Mahidol University, Nakhon Pathom 73170, Thailand; chayanan.boo@student.mahidol.ac.th; 2 Institute for Innovative Learning, Mahidol University, Nakhon Pathom 73170, Thailand; supan.yod@mahidol.edu; 3 Institut des Molécules et Matériaux du Mans, IMMM—UMR CNRS 6283, Le Mans Université, CEDEX 09, 72085 Le Mans, France; Lazhar.Benyahia@univ-lemans.fr

**Keywords:** silica aerogel, natural rubber latex, thermal insulation, insulation materials, latex mixing

## Abstract

In this work, the novel natural rubber latex (NRL) mixing was approached. The mixing process was carried out by using n-hexane as the dispersed phase of silica aerogel which acted as thermal insulation filler prior to NRL mixing. The silica aerogel/NR composites were prepared with different silica aerogel contents of 20, 40, 60, 80, and 100 parts per hundred rubber (phr). The morphology of the 40 phr composite showed the NR macropore formation with silica aerogel intercalated layers. The optimal content of silica aerogels and n-hexane were the key to obtaining the NR macropore. The thermal insulation performance of silica aerogel/NR composites was investigated because of their porous structures. The thermal conductivity of the composites were lower than that of the neat NR sheet and decreased from 0.081 to 0.055 W m^−1^·K^−1^ with increasing silica aerogel content. The lower densities of the composites than that of the NR sheet were revealed noticeably. In addition, the silica aerogel/NR composites exhibited a higher heat retardant ability than that of the NR sheet, and the comparable glass transition temperatures (T_g_) of the composites and the neat NR indicated the maintained flexibility at ambient temperature or higher, which can benefit various temperature applications. The overall results demonstrated that the silica aerogel/NR composites from the novel NRL mixing preparation could be a promising technique to develop the porous materials and be utilised as thermal insulation products and building constructions.

## 1. Introduction

Natural rubber latex (NRL), a direct product from rubber tree (*Hevea brasiliensis* tree), is a milky colloid consisting of poly(1,4-cis-isoprene), water, and non-rubber parts such as proteins, fatty acid, phospholipids, carbohydrates, etc. [1,2,3]. Its outstanding properties are such as high elasticity and good film formation bringing about many applications with the latex mixing method [4,5]. The general concept of latex mixing method is the polymers or additives need to be dispersed in aqueous medium before suspending to the latex, because NRL is the NR particles dispersed in water. Normally, the pre-suspended additives or fillers in aqueous medium method are carried out by applying complicated dispersion procedures such as ball mill, ultrasonication or high-speed mechanical stirring, especially with the addition of surfactant for hydrophobic fillers to obtain the desired dispersion [6]. This issue consists of tedious steps which consume time and energy. To develop a simplify idea of premixing additive by using suitable solvent to disperse similar surface chemistry of additives, i.e., suspension of hydrophobic fillers or additives with suitable organic solvent, and mixing directly to NRL become an interesting alternative and challenging approach.

Traditionally, the purpose of organic solvent addition to NRL is the recovery of rubber from the latex, also known as rubber extraction. Generally, hydrocarbon solvents are used in the separation of NR from latex through the solvation mechanism. The suitable hydrocarbon solvents are the four to nine carbon atoms alkane such as heptane, nonane, pentane, and hexane; the cycloalkane having from five to ten carbon atoms such as cyclohexane and cyclopentane; the six to twelve carbon atoms aromatics or alkyl substituted aromatics such as benzene, xylene, and toluene. Cole et al. extracted NR from 10% total solid content NRL by mixing solvent between hexane and acetone; after strong agitation, three phases separated into the upper phase and lower phase of very fluid liquid phase, while the middle phase obtained viscous liquid. This viscous liquid was further extracted and analysed to be NR. Hexane was the solvent in the middle phase yielding viscous swollen gel as hexane acts as the good solvent of NR [7]. The diffusion ability of good solvent provides partial solvation of NR in the mixture. Thereby, in this work, the authors purpose using premixed hydrophobic additives with suitable solvent and mixing with NRL directly as the hydrophobic additives can still move around the NRL through good solvent diffusion.

In addition, the solubility parameter of rubber in NRL with different non-polar organic solvents needs to be considered, because the viscosity of the mixture depends on the viscous phase content in the system. The high viscosity mixture brings about difficult mixing. The proper solvent should create less viscous phase. Therefore, the solubility parameter of natural rubber and different solvent are also used as criteria to select the suitable solvent. Tangboriboonrat et al. reported the lowest different solubility value between NR and solvent showed greatest solvation effect, i.e., expansion of rubber chain. Cyclohexane showed the least different solubility parameters, xylene and toluene, respectively, when using the average solubility parameter of non-crosslink NR was 16.65(J cm^−3^)^1/2^ [8]. Jing et al. determined the total Hildebrand solubility parameter (δ_t_) of NR with 30 different solvents reported that the crosslink NR showed highest swelling trend with aromatics, amines, and cycloalkanes. The linear alkanes and its derivatives such as n-hexane presented the moderate swelling performance. Alcohols and amides were categorised as lower-swelling solvent [9]. n-Hexane became a promising solvent that provide easy mixing with simple mechanical stirring yielding lower viscosity mixture than other solvents. In addition, the low surface energy and the low boiling point of n-hexane make it easy to evaporate and could benefit the drying procedure [10].

In this work, silica aerogels were used as the hydrophobic additive to improve the thermal insulation performance of NR. Silica aerogels are remarkable as excellent thermal insulation material due to their physical structures presenting a high specific surface area, high porosity of 99% which contribute to extremely low density, and lowest thermal conductivity of 0.01–0.03 W m^−1^·K^−1^ [11,12,13,14,15,16,17]. The NR composite was prepared via a novel latex mixing method that was a new challenge to reduce the preparation time and use of complicated instruments. Silica aerogels were premixed with n-hexane prior to mixing with NRL. The effect of the preparation procedure on the composite morphologies was investigated by FESEM. The thermal conductivity of composites showed a significant decrease from that of neat NR. The apparent insulation performance of the composites showed better efficiency and had lower density.

## 2. Materials and Methods

### 2.1. Materials

Hydrophobic silica aerogel powder was purchased from REM Tech Co., Ltd. (Daejeon, Republic of Korea), and the particle size, surface area, and bulk density were 10–200 nm, 300–350 m^2^ g^−1^, and 70–150 kg m^−3^ respectively. n-Hexane (with a purity of 98.0%) was purchased by RCI Labscan Limited (Bangkok, Thailand). NRL was purchased from Thai Rubber Latex Corporation, Public Company Limited (Samutprakarn, Thailand), and its total solid content (TSC) was approximately 61.75%.

### 2.2. Silica Aerogel/NRL Composite Preparation

Silica aerogel/NRL composites were prepared by adding 10.0%wt diluted NRL to premixed silica aerogel in n-hexane using silica aerogel contents of 20, 40, 60, 80, and 100 parts per hundred rubber (phr). The mixtures were vigorously stirred at 500 rpm for 5 min. Then, each slurry was poured into a glass Petri-dish and left at room temperature for n-hexane to evaporate for about 30 min, then dried in an oven at 60 °C for 2 days. NR sheet as a control specimen was prepared by casting NRL on glass Petri-dish and dried at 60 °C.

### 2.3. Characterisation of the Silica Aerogel/NRL Composite Insulation Properties

Cross-sectional morphologies of the composite were observed with a field emission scanning electron microscope (FESEM) SU 8000, Hitachi (Tokyo, Japan). The specimens were fractured in liquid nitrogen, then coated with a thin layer of platinum to prevent charging under the electron beam. FESEM micrographs were obtained under an accelerated voltage of 10 kV.

The thermal conductivity (k) of the composite specimens was determined by using a thermal conductivity analyser C-Therm TCi, C-Therm Technologies Ltd. (New Brunswick, Canada). The instrument is operated based on the Modified Transient Plane Source technique. An electrical current is applied to the heating part of the sensor to generate a small amount of heat, the generated heat causes the temperature to rise by approximately 2 °C at the interface between the sensor and the composite. The temperature rise at the interface induces a voltage drop in the sensor element. The thermal conductivity was determined by measuring the rate of increase in the sensor voltage. The experiment was conducted at room temperature. Thermal insulation property of the composite is indicated by the lower thermal conductivity value measured. Each specimen was tested with three different runs [18,19].

The density of composite was measured by the hydrostatic method (ASTM D 297) with Densimeter MD-200S, SP Metrology System Co. Ltd. (Pathumthani, Thailand). The density at 25 °C was obtained by weighing the specimen in air, then, a very fine wire used to prevent the sample from floating was placed on the water surface in the water tank, then carefully placed the specimen cautiously under the wire not to create bubbles that would cause errors. Subsequently, the specimen was weighed under the water. Each specimen was measured five times to eliminate statistical errors. The density was calculated by the equation as follows:

(1) $$\mathrm{Density}\;\mathrm{at}\;25\;{}^{\circ}C\;\mathrm{in}\;\mathrm{kg}/m^{3}=\frac{0.9971\;\times \;A}{A\;-\;(B\;-\;C)}$$

where A = mass of specimen in air (g); B = mass of specimen and supporting wire in water (g); and C = mass of supporting wire in water (g).

**Figure 1:**
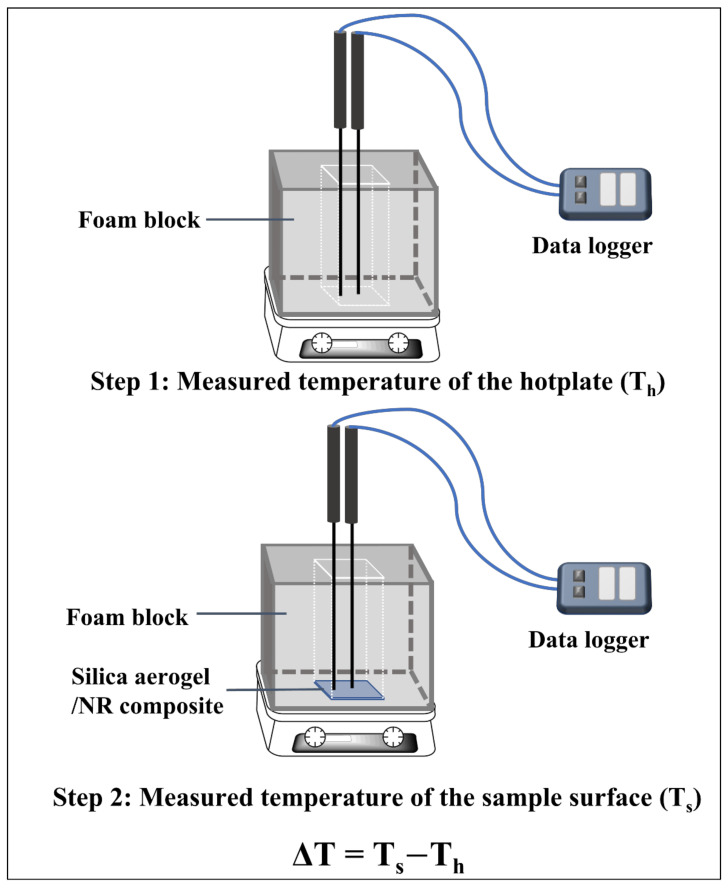
The custom-designed setup to evaluate the apparent insulation performance of the NR sheet and silica aerogel/NR composite with silica aerogel contents.

The apparent insulation performance of the composites was investigated by using a hotplate equipped with a 4-inch thick foam chamber setup, as shown in Figure 1. The hotplate was set to approximately 65 °C. Each specimen was cut into 5 × 6 cm and was 0.267 ± 0.028 cm thick. The data logger was used to measure two positions on the specimen surface. Each specimen was tested at least three times.

Flexibility of composite was determined by the glass T_g_ using the differential scanning calorimeter (DSC) TA Instruments Q200. The specimens (circa 10 mg) were encapsulated in TZero aluminum pans, and the specimens were pre-heated at a heating rate of 20 °C min^−1^ and held at 200 °C for 1 min, then quenched to −80 °C at a rate 10 °C min^−1^. The DSC scan was observed from −80 to 70 °C at a heating rate of 10 °C min^−1^ in nitrogen atmosphere. The T_g_ of each sample was taken as the midpoint of the transition.

## 3. Results and Discussion

### 3.1. Morphology

#### 3.1.1. Understanding the Effect of Silica Aerogel and n-Hexane on NRL Mixing

**Figure 2:**
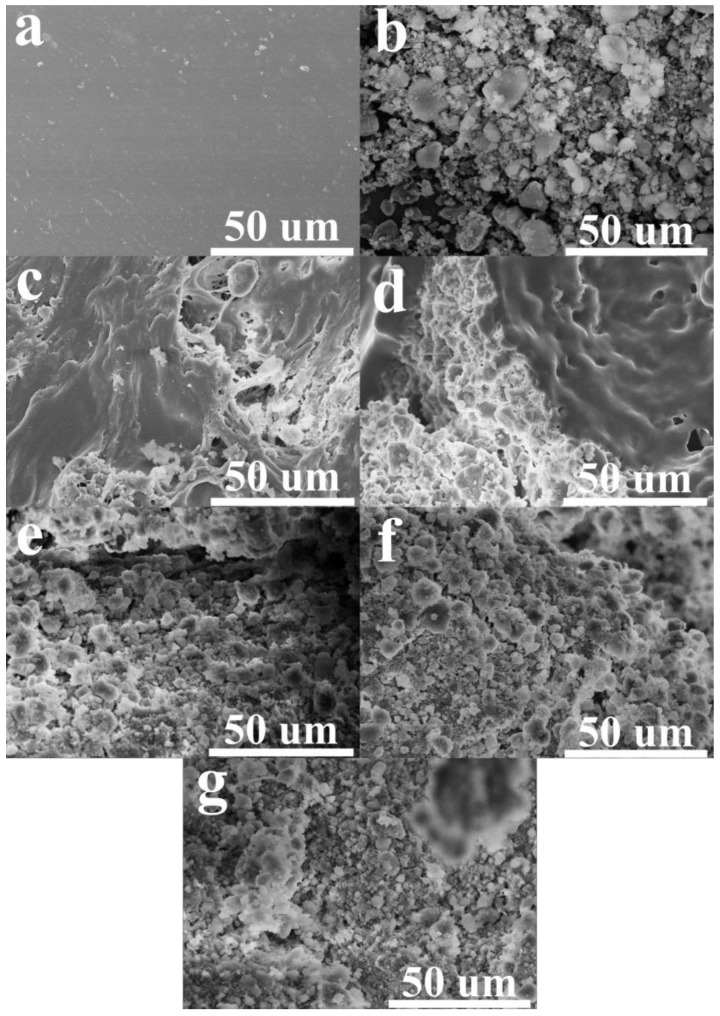
Cross-section FESEM micrographs of (**a**) NR sheet, (**b**) silica aerogel, and silica aerogel/NR composites with (**c**) 20 phr, (**d**) 40 phr, (**e**) 60 phr, (**f**) 80 phr, and (**g**) 100 phr silica aerogel.

Silica aerogel/NR composites were prepared via premixed silica aerogel with n-hexane before latex mixing. The dried composites were used to study the effect of n-hexane and silica aerogel on the composite preparation. The cross-sectional morphologies of the silica aerogel/NR composite included neat NR and pristine silica aerogel, as shown in Figure 2. The neat NR sheet (Figure 2a) had a smooth and dense structure, while the pristine silica aerogel (Figure 2b) showed furry granulated morphology. Figure 2c, d shows silica aerogel/NR composites with both NR and silica aerogel phases for 20 phr and 40 phr silica aerogel content, respectively. The heterogeneous silica aerogel dispersion in the NR matrix was presented in the 20 phr composite. For 40 phr composite silica aerogels were located between the holes of NR phase that presented an NR pore structure with embedded silica aerogel. On the other hand, the NR phase disappeared in the composite with the 60, 80, and 100 phr silica aerogel composites, as shown in Figure 2e–g, respectively. The silica aerogel became the major phase in these composites. According to the different morphologies of the composites with different silica aerogel contents, it is important to understand what parameters affect the composite formation.

**Figure 3:**
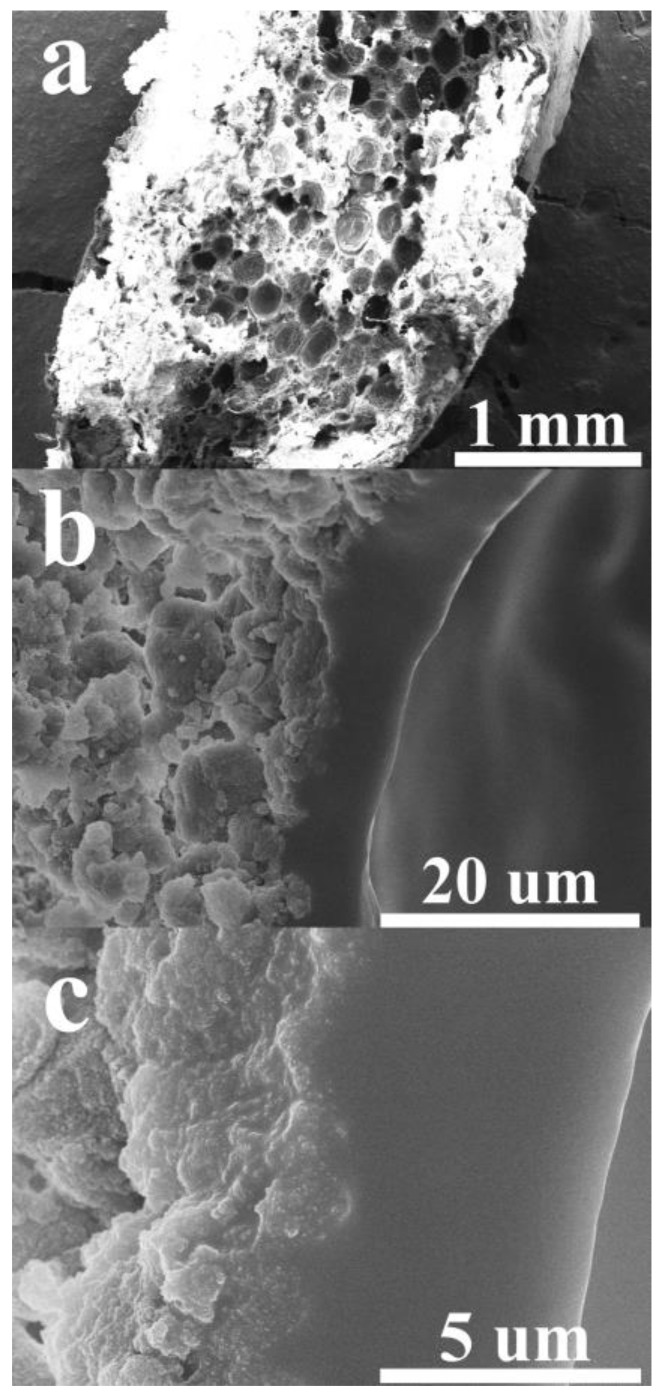
Cross-section FESEM micrographs of silica aerogel/NR composites with different magnifications as (**a**) × 35, (**b**) × 2.5 K, and (**c**) × 10 K.

**Figure 4:**
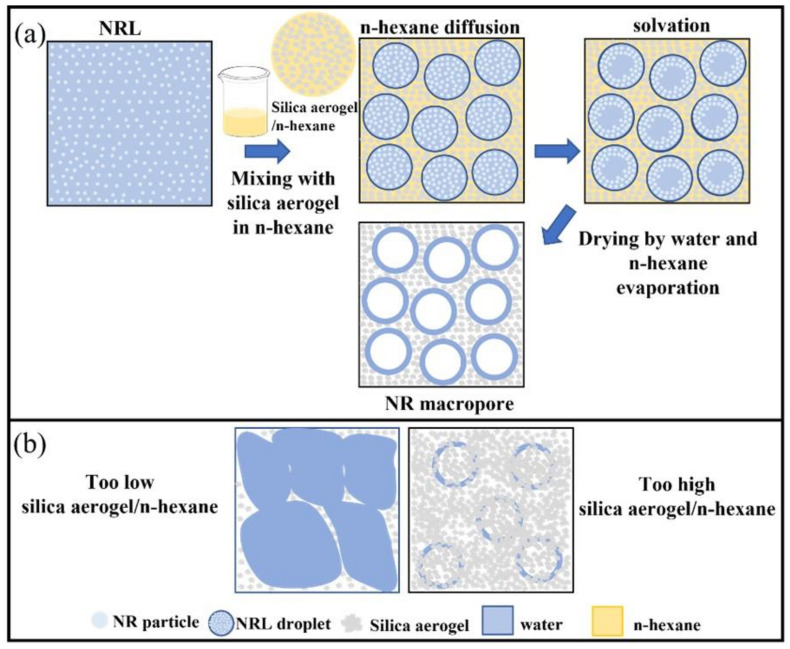
Schematic representation of (**a**) NR macropore formation in silica aerogel/NR composite and (**b**) effect of silica aerogel content on NR macropore.

In the preparation system, NRL content was kept constant with different silica aerogel amounts pre-dispersed in n-hexane. This means that the n-hexane content in the system depended on the silica aerogel content. Hence, the effects of silica aerogel and n-hexane content on the composite formation were discussed. According to the composite morphologies with different silica aerogel content, only the 40 phr composite showed a porous structure. Figure 3 shows different magnifications of the 40 phr composite. The whole composite morphology showed a porous structure in Figure 3a, the silica aerogel phase existed intercalating between the boundary of the pores which showed only the NR phase as shown in Figure 3b. There was silica aerogel dispersing in the NR matrix at the interphase shown in Figure 3c. These phenomena can be ascribed by the solvation mechanism, because n-hexane can dissolve NR that is composed of poly(1,4-cis-polyisoprene) and results in the NRL droplet dispersed in the n-hexane medium during the dissolving process with the existence of the silica aerogel (Figure 4a). The aqueous component in NRL droplets caused the specific dissolving area of NR particles by n-hexane at the droplet surface. This partial NR dissolution was the carrier to disperse silica aerogel to the latex matrix. At the drying process with rapid evaporation of n-hexane, there was still the existence of silica aerogel surrounding NRL droplet resulting in a well-impregnated silica aerogel in partially dissolved NR interphase. Additionally, when the water evaporated from the NRL droplets, it left the NR macropores.

In the case of too low silica aerogel content which also meant too low n-hexane (Figure 4b), the structure of the silica aerogel intercalated layer was too thin to hold the NRL droplet structure and broke-off. Therefore, NRL droplet was not observed for 20 phr. When there was too high silica aerogel, for 60, 80, and 100 phr, the intercalated aerogel was too thick together with lower NR content, the NR phase was not observed in the FESEM micrographs. Therefore, the dried composite only showed the silica aerogel components. It can be concluded that the optimum condition to obtain the porous structure required that n-hexane had to sufficiently diffused throughout the NRL content and formed stable NRL droplets. In addition, the silica aerogel content had to be adequately intercalated between the NRL droplets until the composite dried.

#### 3.1.2. Morphology of Silica Aerogel Pores in Composite

**Figure 5:**
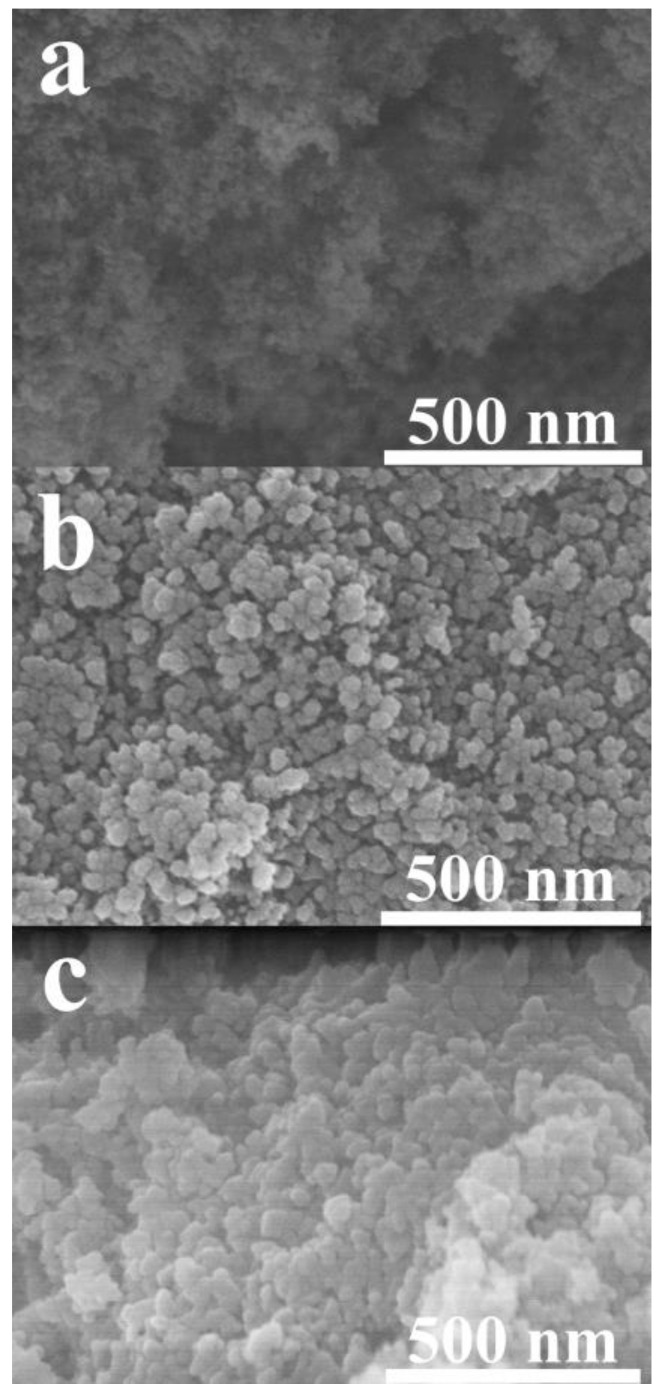
Cross-section FESEM micrographs of high magnification of silica aerogel porous structure as (**a**) pristine silica aerogel, (**b**) silica aerogel in 40 phr composite, and (**c**) silica aerogel in 60 phr composite.

According to the excellent thermal insulation properties of silica aerogel, the nanoporous structure of silica aerogel was studied. Many works reported that the nanopore of silica aerogel is the main role to reduce the thermal conductivity of the bulk composite. Lee et al. [20], Kim et al. [21], and Kim et al. [22] prepared silica aerogel composite via the preservation of silica aerogel nanopores through premixed silica aerogel with low surface tension solvent. This method provided easy evaporation of the solvent without the collapsing of the silica aerogel porous structure prior to the polymer compounding, and obtained super-thermal insulation materials. Figure 5 compares the nanoporous structure of pristine silica aerogel (Figure 5a) and silica aerogel in the composites (Figure 5b,c) where the nanoporous structures were still observed. The silica aerogel nanopores of 40 phr composite (Figure 5b) showed both NR and silica aerogel phases. The silica aerogel nanopores in the composite decreased. The silica aerogel particles were denser than that of pristine silica aerogel. Figure 5c shows the silica aerogel structure of 60 phr composite that had similar results with 40 phr that was reduce nanopores and denser particles. These silica aerogel nanopores obtained were similar to the morphologies reported by Lee et al. using ethanol as a solvent to preserve silica aerogel nanopores and obtaining superinsulation material [20]. The mixing process of 40 phr proved to provide a porous structure of the composite and still obtaining silica aerogel with the nanoporous structure that is still within the range of thermal insulation material.

### 3.2. Thermal Conductivity

**Figure 6:**
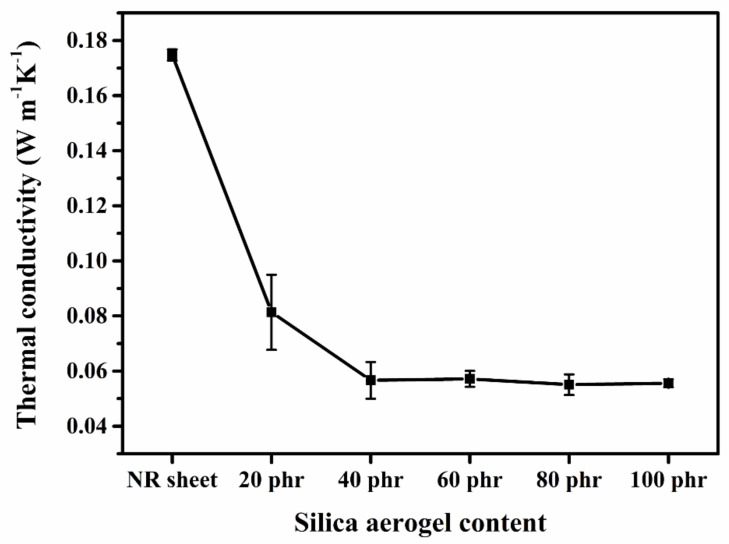
Thermal conductivity of silica aerogel/NR composites in different silica aerogel contents and NR sheet.

Thermal conductivity (k) is a significant parameter that indicates the insulation performance of the thermal insulation materials. The silica aerogel/NR composites with different silica aerogel contents and the NR sheet as control sample were investigated, and the results are shown in Figure 6. According to the results, the k-value of the NR sheet at 25 °C was 0.174 W m^−1^·K^−1^, while the k-value of silica aerogel/NR composite decreased with the increasing silica aerogel content. The k-value of the silica aerogel/NR composite was decreased sharply to 0.056 W m^−1^·K^−1^ in 20 and 40 phr silica aerogel composites. The k-value of 60, 80, and 100 phr silica aerogel composite slightly decreased to 0.055 W m^−1^·K^−1^. The results presented that the composite with the highest silica aerogel content showed 68.19% decrease of the k-value from that of the NR sheet. Hence, because of the presence of the composite pores and silica aerogel nanopores caused the decrease of the k-value. It was reported that the heat transfer by convection is negligible when the pore diameter is lower than 4 mm [23]. It can be ascribed that the NR macropore in the composite showed ca. 0.165 mm pore diameter and the diameters of the silica aerogel nanopores were in the nanoscale. These structures resulted in the reduced of heat transfer by convection. Moreover, the increasing of silica aerogel content in the morphologies of 60, 80, and 100 phr composites caused the disappearance of the NR macropore. The k-value then depended on the nanopores of the silica aerogel, and because the nanopore structures of the composites greater than 40 phr were similar, therefore, only slight reductions of the k-value were noted.

### 3.3. Density

**Figure 7:**
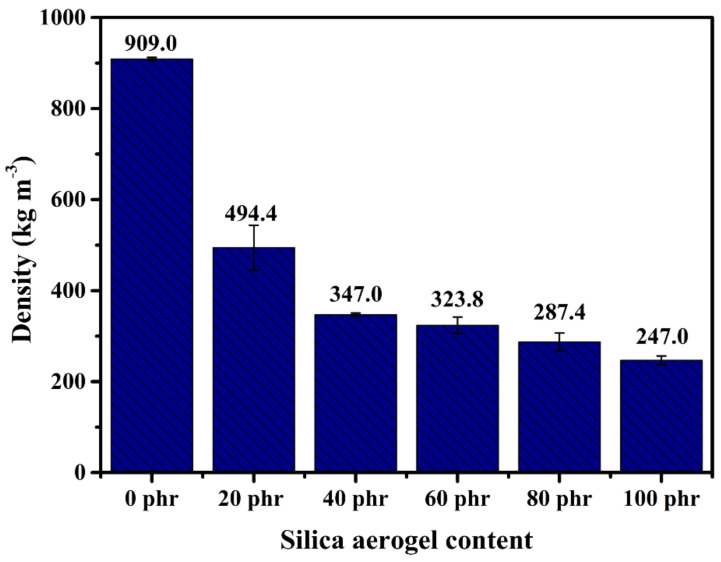
Density of silica aerogel/NR composites in different silica aerogel contents and NR sheet.

The density of the silica aerogel/NR composite was measured by the ASTM D 297 standard (Pennsylvania, United States) by the hydrostatic method. The density is a significant parameter showing foam texture which relates to the porous structure of the composite which brings about the decreasing of the thermal conduction and the thermal convection. The density of the silica aerogel/NR composites is presented in Figure 7. The density of the NR sheet was 909.0 kg m^−3^. The densities of the silica aerogel/NR composites were noticeably lower than that of the NR sheet as 494.4 kg m^−3^ to 247.0 kg m^−3^ with increasing silica aerogel content. The density of 20 phr composite was decreased roughly by 45%, and for 40 to 100 phr composites, the densities decreased by more than 60% compared to the pristine NR sheet.

There was a significant change in the density of the 40 phr composite due to the presence of the NR macropores. The density of 60 phr and higher decreased gradually because there were mainly silica aerogels in the structure. The density of the composites depended on the decreasing NR content. In addition, the results not only corresponded well with the morphologies, but also the thermal conductivities. The density of 20 phr to 40 phr composites decreased significantly similar to the k-values which can be described by the morphology. There was a slightly different trend between the k and the density for 60 phr composites onward. The density decreased gradually while the k-values were constant. This can be ascribed that the density of 60 phr and higher depended on the decrease of the NR content, while the k-values depended on the nanoporous structure silica aerogel that was similar to the morphology of 40 phr composite. The density of the composite confirmed the existence of the porous structure and that the novel mixing can produce material for thermal insulation application.

### 3.4. Apparent Insulation Performance

**Figure 8:**
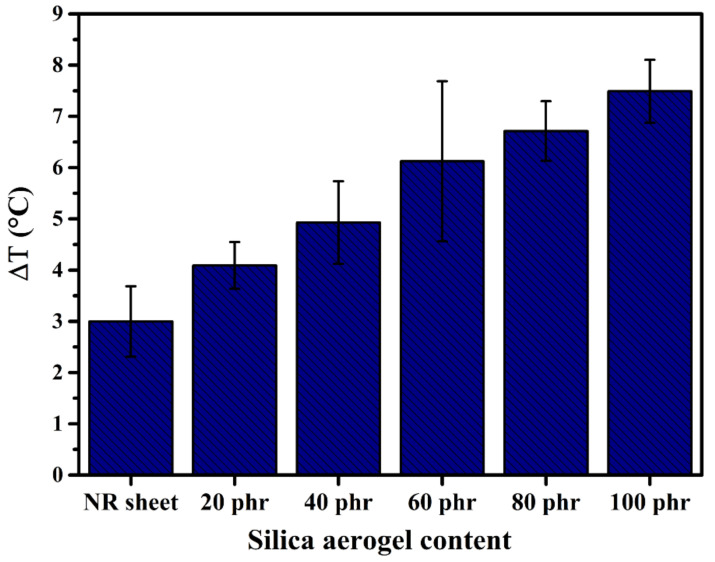
Temperature difference between the temperature of the hotplate and sample surface of silica aerogel/NRL composites in different silica aerogel contents and NR sheet.

The apparent insulation performance of the NR sheet and the silica aerogel/NR composites with different silica aerogel contents was investigated by the temperature difference (ΔT) between the temperature of the hotplate (T_h_) and the sample surface (T_s_). Figure 8 displays the ΔT of all samples that showed the neat NR sheet provided roughly 3 °C retarding the heat source. The composites possessed retarding performance of 4.09 to 7.49 °C with increasing silica aerogel content. These results exhibited that the composites were higher efficiency to retard heat than that of the NR sheet as over 4 °C at the maximum silica aerogel content. The results confirmed that the composites provided inferior thermal insulation performance than pristine NR.

### 3.5. DSC

**Figure 9:**
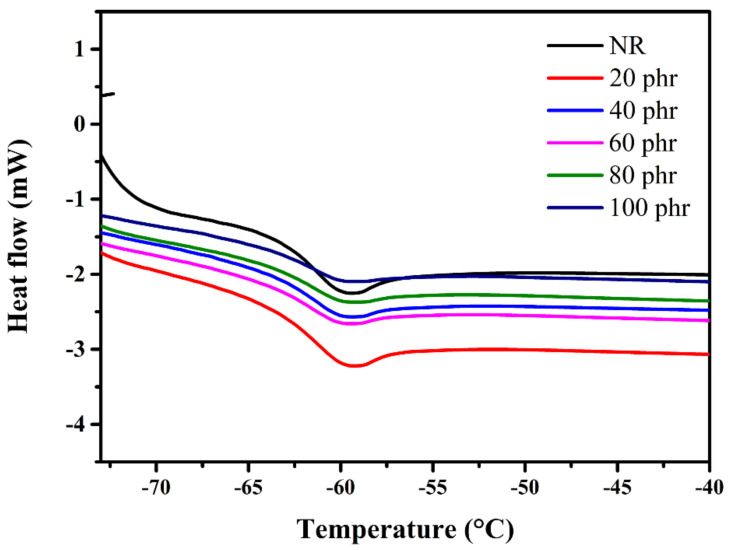
DSC thermogram of silica aerogel/NR composites in different silica aerogel contents and NR sheet.

The T_g_ is an important physical property of polymer, i.e., when the environment temperature approaches T_g_, a transition state of amorphous polymer changes from glassy states to rubbery states [24]. In terms of chain structure, this temperature provides relaxation of the rigid chains turning to the movable chains [25,26]. DSC thermogram (Figure 9) of the neat NR and silica aerogel/NR composites show that the T_g_ of the composites was ca. −61 °C which is similar to T_g_ of the NR sheet. The indistinguishable T_g_ of the neat NR and their composites revealed no significant filler effect on the T_g_ of composites [26]. Zhang et al. prepared NR composite using cellulose nanocrystals as fillers had reported that filler did not affect the T_g_ of NR composites [27] In addition, the T_g_ of the composites showed extremely below than room temperature due to intrinsic T_g_ of the NR that contributed to the composite ability to retain flexibility at room temperature or higher [20].

## 4. Conclusions

To summarise, the novel NRL mixing was prepared successfully by premixed n-hexane and silica aerogel prior to NRL addition. The silica aerogel/NR composites with 20 to 100 phr silica aerogel content were prepared and used to study the composite formation and their thermal insulation performance. The morphology of the composites was used to understand the effects of silica aerogel and n-hexane on composite formation. The 40 phr composite morphology showed the NR macropores with silica aerogel intercalated layers. This structure formation was described by the solvation of n-hexane and NR. The NRL droplets were observed when silica aerogel and n-hexane diffused and turned into NR macropores by evaporation of the aqueous phase. The generation and preservation of the NRL droplet were the key to obtain NR macropore in the composite. The sufficiency of n-hexane generated NRL droplets by diffusing throughout the NRL. The NRL droplets were preserved until the composite dried by the adequate silica aerogel intercalation between NRL droplets. The too low silica aerogel content presented heterogeneous silica aerogel dispersing in NR matrix, because there was a too thin intercalated silica aerogel layer between the NR droplets. The too high silica aerogel content presented too thick silica aerogel layer and with lower NR content resulting in only the existence of the silica aerogel structure. In addition, the nanoporous structure of silica aerogel in the composite was still within the range of thermal insulation material. The thermal conductivity (k) of the composites decreased from 0.081 to 0.055 W m^−1^ K^−1^ with increasing silica aerogel content because of their macropores and silica aerogel nanopores, which was a 68.19% decrease from that of the neat NR sheet. The silica aerogel/NR composite exhibited the density decreasing from 494.4 to 247.0 kg m^−3^ because of the macropore structures and the decreasing NR content. The apparent thermal insulation performance of the composites showed the increased temperature difference from 4.09 to 7.49 °C with increasing silica aerogel contents. The silica aerogel/NR composite flexibility was retained at room or higher temperatures that referred to various temperature utilisations.

## Data Availability

Not applicable.

## References

[1] Nawamawat K., Sakdapipanich J.T., Ho C.C., Ma Y., Song J., Vancso J.G. (2011). Surface nanostructure of *Hevea brasiliensis* natural rubber latex particles. Colloids Surf. A Physicochem. Eng. Asp..

[2] Sansatsadeekul J., Sakdapipanich J., Rojruthai P. (2011). Characterization of associated proteins and phospholipids in natural rubber latex. J. Biosci. Bioeng..

[3] Lim H.M., Misni M. (2016). Colloidal and rheological properties of natural rubber latex concentrate. Appl. Rheol..

[4] Vo M.L., Plank J. (2018). Evaluation of natural rubber latex as film forming additive in cementitious mortar. Constr. Build. Mater..

[5] Cai H.H., Li S.D., Tian G.R., Wang H.B., Wang J.H. (2003). Reinforcement of natural rubber latex film by ultrafine calcium carbonate. J. Appl. Polym. Sci..

[6] Tang L.-C., Zhao L., Qiang F., Wu Q., Gong L.-X., Peng J.-P., Yaragalla S., Mishra R.K., Thomas S., Kalarikkal N., Maria H.J. (2019). Chapter twelve—Mechanical properties of rubber nanocomposites containing carbon nanofillers. Carbon-Based Nanofillers and Their Rubber Nanocomposites.

[7] Cole W.M., Bohm G.G.A., Tomaszewski W., Huang Y. (2014). Processes for recovering rubber from natural rubber latex. U.S. Patent.

[8] Tangboriboonrat P., Suchiva K., Kuhakarn S. (1994). Characterization of non-crosslinked natural rubber latex by phase transfer technique. Polymer.

[9] Jing Y., Cui Z., Zou H., Tu J., Jiang X., Shi X., Yong Z., Liu S., Liu G. (2022). Three-dimensional solubility parameters of natural rubber and its predictive power in diffusion coefficients. J. Appl. Polym. Sci..

[10] Wang X., Chen Z., Sun H., Yin Y., Huan Y., Yang X. (2020). Wet mixing with organic solvent for synthesized cis-1,4-polyisoprene-based rubber composites. ACS Omega.

[11] Lee C.J., Kim G.S., Hyun S.H. (2002). Synthesis of silica aerogels from waterglass via new modified ambient drying. J. Mater. Sci..

[12] Hüsing N., Schubert U. (1998). Aerogels—Airy materials: Chemistry, structure, and properties. Angew. Chem. Int. Ed..

[13] Xie T., He Y.L. (2016). Heat transfer characteristics of silica aerogel composite materials: Structure reconstruction and numerical modeling. Int. J. Heat Mass Transf..

[14] Wang X.D., Sun D., Duan Y.Y., Hu Z.J. (2013). Radiative characteristics of opacifier-loaded silica aerogel composites. J. Non Cryst. Solids..

[15] Yan J., Choi H.Y., Hong Y.K., Jeong Y.G. (2018). Thermal insulation performance of cotton and PET-based hybrid fabrics impregnated with silica aerogel via a facile dip-dry process. Fiber Polym..

[16] Saoud K.M., Saeed S., Bertino M.F., White L.S. (2018). Fabrication of strong and ultra-lightweight silica-based aerogel materials with tailored properties. J. Porous Mater..

[17] Saeed S., Soubaihi R.M.A., White L.S., Bertino M.F., Saoud K.M. (2016). Rapid fabrication of cross-linked silica aerogel by laser induced gelation. Microporous Mesoporous Mater..

[18] Kim H.M., Noh Y.J., Yu J., Kim S.Y., Youn J.R. (2015). Silica aerogel/polyvinyl alcohol (PVA) insulation composites with preserved aerogel pores using interfaces between the superhydrophobic aerogel and hydrophilic PVA solution. Compos. Part A..

[19] Do N.H.N., Tran V.T., Tran Q.B.M., Le K.A., Thai Q.B., Nguyen P.T.T., Duong H.M., Le P.K. (2021). Recycling of pineapple leaf and cotton waste fibers into heat-insulating and flexible cellulose aerogel composites. J. Polym. Environ..

[20] Lee H., Lee D., Cho J., Kim Y.O., Lim S., Youn S., Jung Y.C., Kim S.Y., Seong D.G. (2019). Super-insulating, flame-retardant, and flexible poly(dimethylsiloxane) composites based on silica aerogel. Compos. Part A Appl. Sci. Manuf..

[21] Kim H.M., Kim H.S., Kim S.Y., Youn J.R. (2015). Silica aerogel/epoxy composites with preserved aerogel pores and low thermal conductivity. e-Polymers.

[22] Kim S.Y., Noh Y.J., Lim J., You N. (2014). Silica aerogel/polyimide composites with preserved aerogel pores using multi-step curing. Macromol. Res..

[23] He Y.L., Xie T. (2015). Advances of thermal conductivity models of nanoscale silica aerogel insulation material. Appl. Therm. Eng..

[24] Edjenguele A., Alegria A., Arrese-Igor S., Ehabe E.E., Nkengafac N.J. (2021). Preparation and characterization of non-vulcanized natural rubber-based cocoa pod husk composites. J. Appl. Polym. Sci..

[25] Loadman M.J.R. (1985). The glass transition temperature of natural rubber. J. Thermal Anal..

[26] Ginting E.M., Bukit N., Saragih M.T., Frida E., Bukit B.F. (2020). Analysis of natural rubber compounds with filler of oil palm empty bunches powder and carbon black. J. Phys. Conf. Ser..

[27] Zhang C., Dan Y., Peng J., Turng L.S., Sabo R., Clemons C. (2014). Thermal and mechanical properties of natural rubber composites reinforced with cellulose nanocrystals from Southern Pine. Adv. Polym. Technol..

